# Case Report: Severe *Plasmodium vivax* Malaria after Splenectomy

**DOI:** 10.4269/ajtmh.23-0147

**Published:** 2023-06-20

**Authors:** Noy Norman Kambuaya, Hasrini Rini, Putu Ayu Indra Shanti, King Alexander, Freis Candrawati, Pak Prayoga, Leo Leonardo, Dewi Sri Margayani, Bagus Tesa Gina Yayang, Enny Kenangalem, Pierre A. Buffet, Nicholas M. Anstey, Jeanne Rini Poespoprodjo, Steven Kho

**Affiliations:** ^1^ Timika Malaria Research Facility, Papuan Health and Community Development Foundation, Timika, Papua, Indonesia;; ^2^ Rumah Sakit Umum Daerah Kabupaten Mimika, Timika, Papua, Indonesia;; ^3^ Institut Pasteur, University of Paris, Paris, France;; ^4^ Global and Tropical Health Division, Menzies School of Health Research and Charles Darwin University, Darwin, Australia;; ^5^ Department of Pediatrics, Gadjah Mada University, Yogyakarta, Indonesia

## Abstract

Severe malaria after splenectomy has been reported with infections with *Plasmodium falciparum*, *Plasmodium knowlesi*, and *Plasmodium malariae,* but is less well-characterized with *Plasmodium vivax*. We describe a case of severe *P. vivax* malaria with hypotension, prostration, and acute kidney injury occurring 2 months after splenectomy in Papua, Indonesia. The patient was treated successfully with intravenous artesunate.

## INTRODUCTION

The role of the spleen in human malaria is complex. In acute malaria, the spleen has a protective role in phagocytic clearance of circulating parasites, including after antimalarial treatment.^1^^,^^2^ The spleen is also the major reservoir for viable *Plasmodium falciparum* and *Plasmodium vivax* parasites, in chronic malaria at least, and the site of cryptic endosplenic life cycles with both species.^3^^,^^4^ Splenomegaly is commonly seen in both acute and chronic malaria, and can lead to hyperreactive malarial splenomegaly in endemic areas after repeated exposure to the parasite. As a secondary lymphoid organ, the spleen also has a key role in systemic immunity to *Plasmodium* through cell-mediated and memory cell mechanisms.

Without a functional spleen, individuals are at increased risk of severe and potentially fatal infections, especially those caused by encapsulated bacteria,^5^ and *Plasmodium* and *Babesia* parasites. Individuals who have undergone a splenectomy have an increased risk of clinical episodes of *P. falciparum* malaria,^6^^,^^7^ with the risk of postsplenectomy malaria in coendemic areas being greater for *P. vivax* than *P. falciparum*.^8^ Patients with *P. falciparum* infections after splenectomy display higher parasitemias and a greater frequency of mature forms in circulation.^9^ Case reports and series also suggest a greater frequency of severe disease and death from *P. falciparum* infection after splenectomy, particularly in nonimmune patients.^2^^,^^9^^,^^10^ Cases of severe malaria after splenectomy are also well described in infection by non-*P. falciparum* species, and have been reported in *Plasmodium knowlesi*^11^^,^^12^ and *Plasmodium malariae*^13^^,^^14^ malaria. Here we report a case of severe *P. vivax* malaria after splenectomy in malaria-endemic Papua, Indonesia.

## CASE REPORT

A 48-year-old male resident of Timika, Papua, Indonesia, underwent splenectomy at Rumah Sakit Umum Daerah (RSUD) Hospital in 2021 for hypersplenism and massive splenomegaly (2.3 kg). He had a history of hepatitis C, but no clinical episodes of malaria in the preceding year. Preoperative pan-*Plasmodium* lactate dehydrogenase (pLDH)/histidine-rich protein 2 (HRP2) and HIV rapid diagnostic tests were negative; however, routine preoperative blood smear revealed asymptomatic peripheral *P. falciparum* ring-stage parasitemia with a density of 443 parasites/μL. This was cleared with 3 days of oral dihydroartemisinin–piperaquine (DHP) and, after exclusion of glucose-6-phosphate dehydrogenase deficiency, a course of supervised primaquine (PQ) with a total dosage of 7 mg/kg per local postsplenectomy guidelines. The patient also received immunization with pneumococcal, meningococcal, *Haemophilus*, and influenza vaccinations upon recovery from splenectomy.

**Figure 1.**
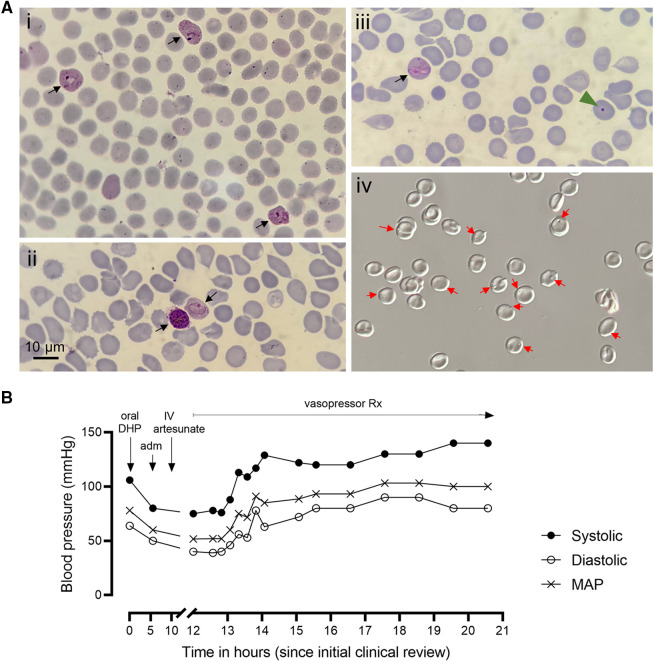
(**A**) Microscopic images from a Giemsa-stained blood film during severe malaria episode are shown (i and ii), indicating the presence of *Plasmodium vivax* asexual stages (6% rings, 91% trophozoites, and 3% schizonts; black arrows) at a density of 12,246 parasites/μL. Absence of splenic function was confirmed by the presence of Howell-Jolly bodies on Giemsa-stained smears (iii; green arrowhead) and an elevated percentage of pocked red cells on wet mounts examined by differential interference contrast microscopy (iv; red arrows). (**B**) Longitudinal blood pressure and mean arterial pressure (MAP) measurements in the first 21 hours after initial clinical review. Vertical arrowheads indicate commencement of oral dihydroartemisinin–piperaquine antimalarials (oral DHP) prior to hospital admission (adm), and commencement of intravenous (IV) artesunate after admission. Horizontal arrow indicates duration of vasopressor treatment, which continued for 28 hours.

During a scheduled clinical review 2 months after splenectomy, the patient was noted to have fever, headache, and myalgia. Vital signs were initially normal (blood pressure, 106/64 mmHg; pulse rate, 75 beats/min; respiratory rate, 24 breaths/min; temperature, 36.3°C; oxygen saturation, 96%). Blood film revealed *P. vivax* asexual stages (6% rings, 91% trophozoites, and 3% schizonts) at a density of 12,246 parasites/μL and low-level gametocytemia (32 parasites/μL) (Figure 1Ai and 1Aii). A pan-pLDH/HRP2 rapid test was positive for pan-pLDH only. He was diagnosed with uncomplicated *P. vivax* malaria and received his first dose of supervised oral DHP and PQ. He did not take his previously prescribed postsplenectomy standby dose of amoxicillin or paracetamol. His condition declined rapidly, and he was admitted to the RSUD Hospital Emergency Department 6 hours later with symptoms of fever, chills, headache, myalgia, nausea, and weakness. On examination, he was unable to sit or stand unaided. He had a fever of 41°C, pulse rate of 120 beats/min, a blood pressure of 80/50 mmHg, a respiration rate of 22 breaths/min, and an oxygen saturation of 96% on room air. His consciousness was normal, with no pallor, icterus, or hepatomegaly. Chest examination was normal. Capillary refill time was <2 seconds. Automated full blood count revealed a hemoglobin level of 12.5 g/dL with 37.2% hematocrit. Platelet (180 × 10^3^/μL), total white cell (7 × 10^3^/μL), and neutrophil counts were all normal. Kidney function testing revealed elevated creatinine (1.7 mg/dL) with normal blood urea nitrogen (12.4 mg/dL). Total bilirubin was normal (0.6 mg/dL) and cell-free hemoglobin was elevated (45,500 ng/mL), both measured retrospectively on frozen heparinized plasma. Liver function tests, and sodium and chloride levels were within normal limits, with mild hypokalemia. Urine output was normal, but further tests were not performed. A SARS-CoV-2 rapid antigen test was negative. During his previous splenectomy, spleen tissue had not been reimplanted, and elevated numbers of pocked red cells (30.8%) and the presence of Howell-Jolly bodies (0.4%) confirmed an absence of splenic function (Figure 1Aiii and 1Aiv). Microscopy 6 hours after commencing outpatient antimalarial treatment confirmed infection with *P. vivax*, although parasitemia was lower (3,432 parasites/μL).

The patient was diagnosed with severe *P. vivax* malaria according to the following modified WHO criteria^15^: hypotension/shock and prostration. He also had stage 2 acute kidney injury (AKI) by Kidney Disease: Improving Global Outcomes (KDIGO) criteria. He was given 500 mL Ringer’s lactate for fluid resuscitation and 1 g of intravenous paracetamol every 6 hours. Parenteral artesunate therapy was given intravenously at a dose of 2.4 mg/kg, which was repeated after 12 and 24 hours. Despite fluid administration, the patient’s blood pressure remained low and dropped to 75/40 mmHg (Figure 1B), prompting the start of 500 mL Ringer’s lactate for a second fluid resuscitation, and intravenous norepinephrine titrated to maintain a mean arterial pressure (MAP) > 80 mmHg. Target MAP was achieved after 2 hours, and vasopressor support continued for 28 hours. Intravenous artesunate was given for 24 hours, followed by 3 days of oral DHP and 15 days of PQ, totaling 7 mg/kg. Antibiotics were not given. Chest examination, respiratory rate, and oxygen saturation remained within normal limits during hospital care. The patient’s hemodynamic status was monitored closely, with the last recorded blood pressure being 140/80 mmHg prior to discharge after 4 days in hospital care. Parasite clearance was confirmed by microscopy at a follow-up 18 days after discharge. A follow-up creatinine concentration 8 months after discharge (0.71 mg/dL) confirmed normalization of his renal function and defined his AKI at the time of severe malaria as KDIGO stage 2 (creatinine 2–2.9 times baseline).

## DISCUSSION

We report a case of severe malaria from *P. vivax* infection after recent splenectomy, characterized by hypotension and prostration, with concomitant KDIGO stage 2 AKI. Postsplenectomy severe malaria caused by infection with *P. falciparum*, *P. knowlesi*, and *P. malariae* is well described, with this case showing that postsplenectomy severe malaria can also be caused by *P. vivax* infection.

Severe anemia has also been reported with *P. vivax* malaria after splenectomy. In both cases, the contribution of *P. vivax* malaria to severe anemia postsplenectomy was confounded by significant preexisting anemia at splenectomy and postulated contributory causes (sickle beta-thalassemia hemoglobinopathy and autoimmune hemolytic anemia, respectively).^16^^,^^17^

Severe malaria, including hypotension, prostration, and AKI, is reported with *P. vivax* malaria in patients with their spleen intact.^18^ Severe sepsis from infection with encapsulated bacteria after splenectomy is also well described,^5^ and concomitant bacteremia with *Salmonella* spp.^19^ and *Streptococcus pneumoniae*^18^ has been reported in *P. vivax* malaria. Absence of microbiology facilities prevented testing for the presence of concomitant bacterial infection contributing to manifestations of severe malaria in this patient, and a chest radiograph was not performed to rule out concomitant bacterial pneumonia. However, because the patient recovered without any antibiotic therapy and improved with antimalarial therapy alone, we think it is likely that the severe clinical manifestations of malaria were the result of *P. vivax* infection alone. Nevertheless, early empirical antibiotics should be given in addition to antimalarial therapy in future patients with *Plasmodium* infection and severe malaria syndrome who have undergone splenectomy to cover the possibility of concomitant life-threatening bacterial infection.

A high frequency of *P. vivax* malaria within 3 months after splenectomy^4^^,^^8^ is well described in this region, which is coendemic for *P. vivax* and *P. falciparum*.^20^ Early recurrence of *P. vivax* malaria after treatment of *P. falciparum* malaria is similarly described in patients with an intact spleen, and is hypothesized to be the result of relapse of preexisting *P. vivax* hypnozoites.^21^ This patient received supervised DHP therapy for his perioperative *P. falciparum* infection, and supervised radical cure with high-dose 7-mg/kg PQ immediately after splenectomy, per local policy, suggesting that the early recurrence of *P. vivax* malaria was not the result of a relapse. Reinfection with *P. vivax* was considered more likely, given his resumption of occupational exposure as a farmer in the lowland forested area of Timika.

In postsplenectomy malaria from *P. falciparum* and *P. knowlesi*, there is no spleen to trap and remove circulating parasites, and circulating parasitemia is greater than in patients with an intact spleen.^6^^,^^11^ Severe disease in spleen-intact *P. vivax* malaria is associated with greater circulating and total parasite biomass, with parasite biomass-related endothelial activation and dysfunction, and with systemic inflammation.^18^^,^^22^ This patient’s circulating parasitemia was in the highest quartile of parasite counts previously seen in patients with an intact spleen hospitalized with *P. vivax* malaria in the same region.^23^ We propose that, with a greater proportion of total *P. vivax* biomass found in the peripheral circulation after splenectomy, endothelial activation and systemic inflammation arising from a relatively high circulating parasitemia may have contributed to disease severity in this patient. In the presence of a spleen, a large proportion of malaria-associated hemolysis occurs in the extravascular compartment, particularly the spleen. In contrast, after splenectomy, most malaria-associated hemolysis occurs in the intravascular component.^11^ This was evident by the elevated plasma cell-free hemoglobin in this patient, in the highest quartile reported previously in patients with an intact spleen hospitalized with *P. vivax* malaria.^22^ We speculate that a greater proportion of intravascular hemolysis led to greater heme-mediated toxicity to endothelial and renal tubular cells than would usually occur in patients with *P. vivax* malaria and an intact spleen,^22^ and likely also contributed to severe disease and AKI in this patient.

In conclusion, this case demonstrates that *P. vivax* infection after splenectomy can also cause severe malaria. The case highlights the importance of rapid diagnosis and treatment of all species causing malaria in hyposplenic populations. Postsplenectomy malaria preventative measures, including chemoprophylaxis, should be considered in areas endemic for *P. vivax* as well as *P. falciparum*.
